# Modeling the NF-κB mediated inflammatory response predicts cytokine waves in tissue

**DOI:** 10.1186/1752-0509-5-115

**Published:** 2011-07-19

**Authors:** Pernille Yde, Benedicte Mengel, Mogens H Jensen, Sandeep Krishna, Ala Trusina

**Affiliations:** 1 Niels Bohr Institute, Blegdamsvej 17, DK 2100, Copenhagen, Denmark

## Abstract

**Background:**

Waves propagating in "excitable media" is a reliable way to transmit signals in space. A fascinating example where living cells comprise such a medium is Dictyostelium D. which propagates waves of chemoattractant to attract distant cells. While neutrophils chemotax in a similar fashion as Dictyostelium D., it is unclear if chemoattractant waves exist in mammalian tissues and what mechanisms could propagate them.

**Results:**

We propose that chemoattractant cytokine waves may naturally develop as a result of NF-*κ*B response. Using a heuristic mathematical model of NF-*κ*B-like circuits coupled in space we show that the known characteristics of NF-*κ*B response favor cytokine waves.

**Conclusions:**

While the propagating wave of cytokines is generally beneficial for inflammation resolution, our model predicts that there exist special conditions that can cause chronic inflammation and re-occurrence of acute inflammatory response.

## Background

Inflammatory response (IR) in higher organisms requires efficient chemotaxis of neutrophils to sites of infection [1]. At the same time excessive neutrophil accumulation has been shown to play a role in diseases such as asthma, atherosclerosis, multiple sclerosis, inflammatory bowel disorder and arthritis [2]. It however remains an open question how the chemoattractant signal is transmitted through the tissue. "Propagating waves" present an optimal way of transmitting a signal across large distances and occur in many biological systems [3], [4]. In particular, propagating waves of chemoattractant are utilized by the social amoeba Dictyostelium D. - a model system for neutrophil chemotaxis [5]. While neutrophils *can *efficiently chemotax through chemoattractant waves [5] it is unclear if they ever encounter such situations. Unlike Dictyostelium, neutrophils do not generate the waves themselves and it remains an open question if there exists a mechanism that could initiate and propagate waves of chemoattractant during IR.

We here suggest that NF-*κ*B is the missing link relating IR in tissue cells to the propagation of a chemoattractant signal. NF-*κ*B upregulates transcription of many cytokines which serve as chemoattractants for neutrophils e.g. TNF, IL-1, IL-6, IL-8 and IL-11 [6-8]. At the same time these cytokines activate NF-*κ*B response. We show that a simple model of spatially coupled tissue cells contains all the necessary components to initiate and propagate waves of chemoattractant cytokines. This model behaves as an "excitable medium" [9] and relies on the following well-known characteristics of IR: 1) fast transient response of NF-*κ*B, 2) a positive feedback from NF-*κ*B to cytokines and 3) short half-life of cytokines. Using mathematical modeling, we find that all these properties favor formation and propagation of cytokine waves.

### Propagating waves - an optimal strategy for signaling in the tissue?

**Figure 1:**
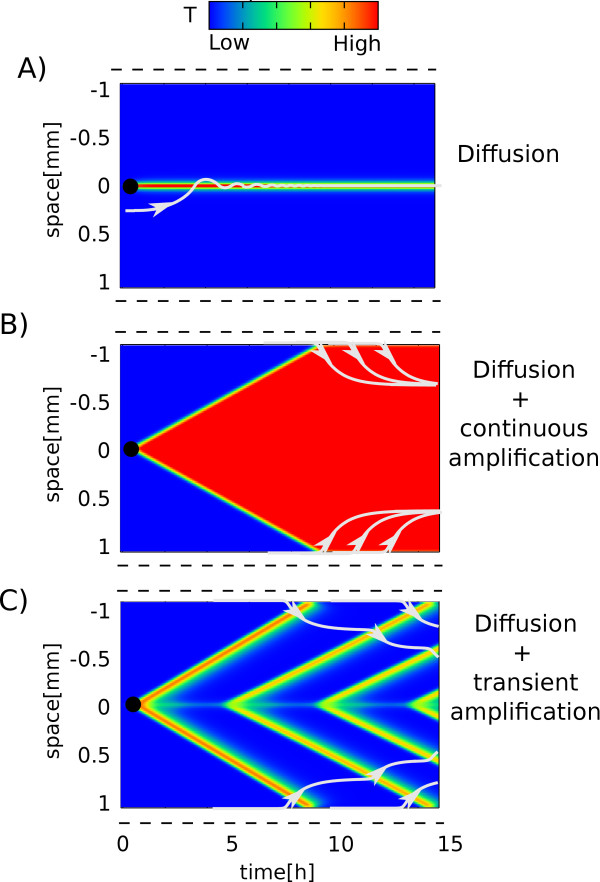
**Three scenarios for signal propagation from the site of infection**. Three scenarios for signal propagation from the site of infection marked by the black circle. Cells are aligned vertically and changes of chemoattractant concentrations in time are developing from left to right. The blue (red) encode low (high) concentrations of chemoattractant (T). White lines represent trajectories of neutrophils chemotaxing from blood vessels (dashed lines). The trajectories were calculated using a "Local Excitation - Global Inhibition" model, described in the additional file 1. In A) the chemoattractant is diffusing from the site of infection. The signal is short-ranged, and no neutrophils are recruited from the distant blood vessels. In both B) and C) the signal is long-ranged. In B) the diffusing chemoattractant is continuously amplified by tissue cells. The gradient is sharp at the blood vessel but it disappears deeper in the tissue thus leaving neutrophils devoid of direction. In C) diffusing chemoattractant is amplified *transiently*. The waves recruit neutrophils from the blood stream and also serve as a directing signal for the neutrophils that are already in the tissue.

In principle there are multiple ways a chemoattractant signal can be transmitted through the tissue, however not all of them are equally efficient and reliable. In the simplest scenario the chemoattractant molecule passively diffuses from the site of infection. This will result in a short-ranged signal where the concentration decays exponentially with the distance from the source (see Figure 1A). The range of the signal will be further limited by the typical short half-life of the chemoattractant molecules.

Some neutrophil chemoattractants, e.g. the cytokines TNF and IL-1, have the unique ability to self-amplify by means of tissue cells or tissue resident macrophages. For example, the upregulation of TNF in a localized area of myocardium can easily induce TNF upregulation in neighboring normal myocardium [10]. This active participation of tissue cells *amplifies *the cytokine concentration across the tissue as illustrated in Figure 1B. Blood vessels serve as a sink for cytokines where they are carried away by the bloodstream. As a result a sharp gradient develops near the blood vessel. It is important to note that both the "diffusion" and the "amplification" (Figure 1A and 1B) scenarios create static gradients, i.e. once in steady state the gradients are not changing in time. Such static gradients are intrinsically toxic for the tissue as, for example, continuous exposure of tissue cells to high TNF levels triggers apoptosis [11]. Interestingly, some experimental evidences suggest that static gradients are also suboptimal for neutrophil chemotaxis; neutrophils seem to orient themselves better when exposed to temporally varying gradients [5,12-15].

If the cytokine concentration is not amplified continuously but *transiently *(i.e. with a peak-like profile), the tissue cells will avoid sustained exposure to toxic cytokines while the signal - the chemoattractant gradient - can still penetrate far in the tissue. Such transient amplification can result in single or re-emerging "propagating waves" as shown in Figure 1C.

It turns out that cytokines are indeed amplified only transiently [16]. Cytokines induce activation of NF-*κ*B - a key regulator of IR in tissue cells. In turn active NF-*κ*B upregulates cytokine production [6-8], thus constituting an amplifying *positive feedback*. If NF-*κ*B-response to inflammatory stimuli is transient, so will be the amplification. And indeed, the synthesis and secretion of inflammatory cytokines from tissue cells were shown to be parallel to the the NF-*κ*B transient activation [16].

## Results and Discussion

### Model

**Figure 2:**
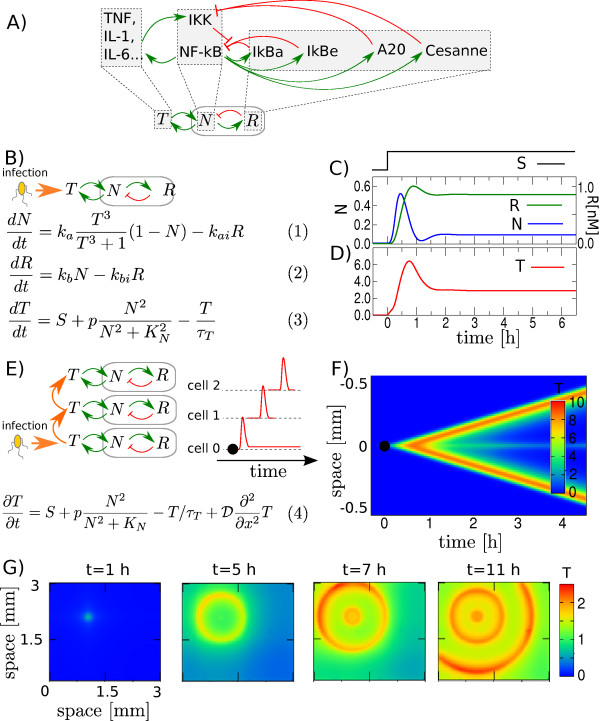
**Model**. Model construction: A) Multiple feedbacks in NF-*κ*B regulatory network are reduced to the core circuit consisting of positive cytokine feedback, *T *→ *N *→ *T*, that allows signal amplification and negative feedback, *N *→ *R *⊣ *N*, that captures NF-*κ*B transient dynamics. B) Mathematical description of the NF-*κ*B-like circuit. N denotes the fraction of active NF-*κ*B; *T *is the fold induction in cytokine concentration and R is a negative regulator of *N*. C) The transient response of N to the stimuli S, and D) corresponding *T *profile in single isolated cell. E) The model of spatially coupled NF-*κ*B-like circuits. The coupling is through the diffusion of cytokines, *T*, and results in "propagating waves " of *T *shown in space-time plot in F) and G). In G) we illustrate the 2D variant of the model simulated with the same parameters.

The model consists of spatially distributed cells each containing NF-*κ*B-like circuits. The circuit in each cell thus contains 3 variables: An NF-*κ*B-like variable N, a *regulator *variable R that combines the effects of all inhibitors in one variable and a cytokine-like variable T see Figure 2A. The key features of our model which we shall explain in more detail below are: 1) slow inhibition due to negative feedback from inhibitors (R), 2) fast amplification due to positive feedback from cytokines (T) and 3) spatial coupling of the NF-*κ*B-like circuits due to diffusion of extracellular cytokines.

#### Transient response through negative feedback

Upon extracellular stimulation the level of active NF-*κ*B increases to reach a peak value after approximately 30 minutes and has decreased again after approximately one hour [17-19]. It has been shown that this *transient *NF-*κ*B response is assured by multiple negative feedback loops where active NF-*κ*B induces expression of its own inhibitors. Some of these inhibitors act directly on NF-*κ*B, e.g. I*κ*B*α, β *and *ε*. Others, as for example the members of A20 family proteins A20 [20] and Cesanne [21] inhibit upstream signaling components at the level of IKK or above.

The main intent with our model is not to capture all the intricacies of the NF-*κ*B system, but rather to focus on the few mechanisms we believe are crucial for linking the transient response of NF-*κ*B to spatio-temporal profiles of cytokines. With that in mind we choose to reduce the multiple nested negative feedbacks comprising the NF-*κ*B regulatory network to a single negative feedback, see Figure 2A and 2B, equations 1 and 2. The main purpose of this negative feedback, *N *→ *R *⊣ *N*, is to reproduce the transient dynamics of NF-*κ*B in response to TNF-stimulation. It is important to note that this part of the model is a phenomenological generalization, i.e. it aims at a simple mathematical reproduction of observed results while not relating to the exact mechanisms. We have tested and confirmed that the main results hold if the nested negative feedbacks are modeled explicitly, see Figure S2 in additional file 1.

#### Amplification through positive cytokine feedback

The positive feedback where nuclear NF-*κB *up-regulates production of cytokines and they in turn induce NF-*κ*B nuclear localization is described by equations 1 and 3 in Figure 2A. The strength of the N→T upregulation is governed by the parameter p, and thus captures the strength of the entire positive feedback (the strength of the T→N upregulation, *k_a _*is fixed, see Methods section). For simplicity we replace the double negative NF-*κ*B activation pathway - where cytokines (e.g., TNF) activates IKK, which in turn inhibits the NF-*κ*B inhibitor - by *T *directly activating *N*. Mathematically this *T *→ *N *activation term is modeled by a Hill function. It encodes an activation *threshold *in NF-*κ*B system, as recently reported by Turner et al. and Tay et al. [19,22].

#### Spatial coupling of single cells

The spatial coupling of cells is schematically shown in Figure 2E. Newly synthesized cytokines are secreted into the extracellular space where they diffuse and induce NF-*κ*B response in neighboring cells. The diffusion of T is described by the diffusion term in equation (4). Note that T is the only variable that diffuses. The variables N and R are bounded inside the individual cells. The blood vessels are placed at the two ends of the line of cells and are modeled as absorbing (open) boundaries. Absorbing boundaries take into account that the cytokines are carried away by the blood flow, thus producing a sink for the variable T. We further assume that the small blood vessels have negligible effect on cytokine diffusion (the results also hold if the small blood vessels are taken into account, see Figure S1 in additional file 1).

In our analysis we assume the parameters estimated for TNF to be characteristic of multiple cytokines constituting the positive feedback loop. Our model consists of 3 variables for each cell and 8 parameters. Among the 8 parameters there is only one that is essentially unconstrained: The strength of positive feedback, p. The other parameters are estimated as described in Methods section.

### Propagating Waves Arise from Spatially Coupled NF-κB-like circuits

The dynamics of a single isolated NF-*κ*B-like circuit is shown in Figure 2C and 2D. The results are obtained by numerical integration of the ordinary differential equations (1)-(3) (Figure 2B). To trigger the response we add an extracellular stimulus S (see equation (3)). Here and in all following simulations the stimulus, *S*, is added at time 0 and is present at all times unless otherwise mentioned. The stimulus S should be thought of as bacterial endotoxin or initiating cytokines secreted by macrophages.

Shortly after stimulus is added the cell responds by increasing the level of active NF-*κ*B (N). Because of the negative feedback from inhibitors (R), NF-*κ*B (N) decreases back to lower values after approximately an hour. The positive feedback from NF-*κ*B (N) amplifies the concentration of cytokines (T) to become many orders of magnitude larger than it would have reached by the small stimulus *S *alone (Figure 2D). Note that the cytokine concentration (T) also peaks on a timescale similar to that of NF-*κ*B.

Interestingly, our model, although simplified, can capture characteristic biphasic response in both the NF-*κ*B-like variable, *N *(Figure 2C, blue line), and the cytokine-like variable, *T *(Figure 2D, red line). Here an acute phase - with a well pronounced peak - is followed by a late phase - where the concentrations are lower than in the acute phase, but are above the pre-stimulus concentrations. In our model the late-phase of the response is entirely due to cytokine positive feedback. This observation agrees well with in-vivo experiments by Han et al., where authors demonstrated that both TNF and IL-1 are required for the late-phase response [16].

When multiple cells are aligned next to each other (in a one-dimensional lattice) a peak in T propagates from cell-to-cell, see Figure 2E and 2F. These results are obtained using equations (1), (2) and (4).

**Figure 3:**
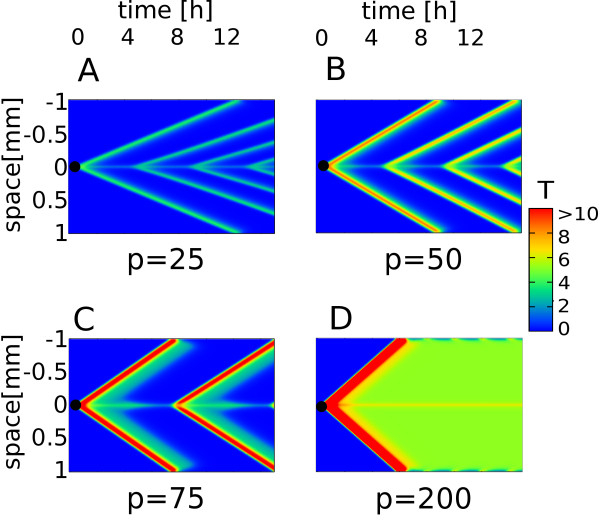
**The role of positive feedback**. Propagating waves re-emerge A)-C) at low to intermediate strengths of positive feedback, *p *= 25, 50, 75. If the amplification is too strong the system is locked in "high T" state, D) *p *= 200.

The main focus of this study is the response to a well localized source of inflammation, i.e. the source of bacterial endotoxin or the cytokine-secreting macrophages accumulated at the location of the damaged cells. We model this by adding a small external stimuli, *S*, at time *t *≥ 0 only to the cell in the middle (Figure 2E, F black circle). The cytokines produced by the stimulated cell at the site of infection will diffuse away and thereby trigger the transient response of the NF-*κ*B system in neighboring cells. The result of this cell-to-cell coupling is a "propagating wave" of NF-*κ*B induction followed by cytokine production and hence a wave of high *T*-concentration propagating through the tissue, see Figure 2F and 2G. Note that the result above relies only on three requirements: 1) Transient response in NF-*κ*B-like variable *N *(slow negative feedback), 2) amplification of the cytokine-like variable *T *(positive feedback) and 3) diffusion of *T *in extracellular space (spatial coupling). Variations in the parameter *p*, show that strong positive feedback generates a more pronounced wave. Both the amplitude and the speed of the wave increase with *p *(see Figure 3).

### Inflammatory response exhibits the characteristics of an "excitable media"

Propagating waves are employed by many other biological systems, which share the need of sending information over relatively large distances, where passive diffusion is insufficient. Similar phenomena are observed in movement of calcium in differentiating Xenopus oocytes [3] and the rapidly propagating action potentials of neurons [4]. These systems, as well as the spatially coupled NF-*κ*B-like circuits, share the properties of an "excitable media". An excitable medium is comprised of locally excitable regions - in our case it is a tissue cell - which all have the ability to get *induced *(excited) and *inhibited*. Such systems are characterized by the "excitation threshold", so that sub-threshold stimuli are rapidly damped, and the system persists in a *resting state *(low *T*, *N *and *R*). Super-threshold stimuli induce sharp local response and the system transits into the *excited state *(high *T *and *N*). Shortly after the response occurs, the region becomes insensitive to further perturbation and is said to be in a *refractory period *(high *R*), after which it can relax back to the resting state where it is again sensitive to perturbations [9].

We have performed a detailed mathematical analysis of the mechanism behind the excitable-media properties of NF-*κ*B-like circuits (see Figure 1S in additional file 1). The analyses confirm that the NF-kB responses coupled in space present a novel example of excitable media.

### Predictions and physiological relevance

It has recently been shown that the circuits combining positive and negative feedbacks allow for robust oscillations [23,24]. We find that our model, where such circuits are coupled in space, can indeed produce *re-emerging *waves.

The model predicts that the conditions for re-establishment depend strongly on two parameters: The strength of cytokine positive feedback, *p *and the cytokine half-life, *τ_T_*. These parameters control the amount of cytokines, *T*, and have to be inversely related, i.e. *p *∞ 1/*τ_T _*to minimize the exposure of tissue cells to cytokines (e.g., a strong positive feedback, that we found to favor wave formation, can be compensated by short cytokine half-life). Remarkably, the reported cytokine half-life is indeed short and ranges between 3-25 minutes [25-27].

**Figure 4:**
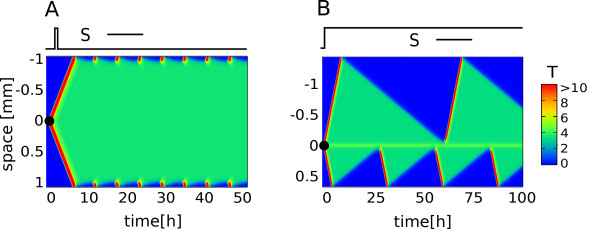
**The effects of absorbing boundaries at the blood vessels**. The effects of absorbing boundaries at the blood vessels. A) Waves persistently re-emerge close to the boundary in response to a short, 30 minutes, pulse of stimuli when *p >*= 105. The group of cells neighboring the source is locked in "high T"- state as shown by green color. B) The frequency of re-initiation is higher if the source is located closer to the absorbing boundary, here *p *= 83.

The analyses of the parameter *p *(Figure 3A-D) show that: 1) The frequency of waves can be modulated by the strength of positive feedback and 2) the dependence is non-monotonic, i.e. the frequency is maximal (corresponding to period of 4.5 hours) at intermediate values of *p *(Figure 3A-D) and 3) there exists a "locked" state with high T concentration and infinite refractory period - see Figure 4D.

The frequency of the cytokine waves can have a direct implication on the amounts of neutrophils recruited to the site of infection (i.e., more frequent waves recruit more neutrophils). As the NF-*κ*B response is modulated at multiple levels (e.g., cytokine receptor desensitization, cooperative transcriptional regulation, etc.) one can imagine a scenario where the strength of positive feedback can be modulated to encode the severity of infection by e.g. increasing the transcription, translation or secretion rates of cytokines.

#### Chronic and recurrent acute inflammation

Surprisingly, the "locked" state with continuously high T (Figure 3D), appears to be self-sustained. Unlike the repetitive waves in Figure 3A-C - which disappear once stimuli is removed - the sustained high production of T can be triggered by just a short pulse of stimuli (Figure 3D and 4A). In the context of IR this situation resembles chronic inflammation - meaning that the response does not resolve even after the damage has been repaired [2].

In Figure 4A it is apparent that the "locked" state is not uniform for all tissue cells. There are roughly two groups of cells: The cells neighboring the source are in a locked state, with T above the excitation threshold. Close to the blood vessels the cytokine concentration will always be low and the nearby cells are able to return to their resting state. At these cells waves can re-emerge, hence creating the situation as in Figure 4A. A similar partitioning of Dictyostelium D. cells into *sustained pacemakers *(the cites where waves originate) at the center of aggregation and *signal transducing cells *elsewhere has been first theoretically predicted by Geberth et al. [28] and later experimentally shown by Gregor et al. [29]. In the case of Dictyostelium D. the state with self-sustained and self-organized pacemakers (which corresponds to "chronic inflammation" state in our model) is desired and so the population works towards reaching high concentration of inducer, i.e. towards a locked state. In the case of NF-*κ*B, the self-sustained pacemakers are undesirable. We expect this system to function at intermediate concentrations of inducer, i.e., a concentration that allows the cells near the cite of infection to be "inducible pacemakers". While these would oscillate in response to the external stimuli, the rest of the cells will propagate the signal (acute response). It is interesting to note that the apoptosis induced by sustained high concentrations of inducer might serve a mechanism that further limits the establishment of self-sustained pacemakers in inflammatory response.

If we consider cytokine waves as a signature for acute inflammatory response and sustained cytokine levels as characteristic of chronic inflammation, then Figure 4A predicts that the two coexist. That is, the chronic inflammation will be accompanied by the recurrent acute IR. Although somewhat contra-intuitive it is frequently observed that, acute and chronic inflammation coexist over long periods, implying continual reinitiation. Examples are found in rheumatoid arthritis, asthma, chronic obstructive pulmonary disease, multiple sclerosis, Crohn's disease, ulcerative colitis, and cancers [2].

Another observation, which is interesting from a physiological perspective is that the frequency of propagating waves can depend on the distance to the boundary for some values of *p *(Figure 4B). The frequency is higher at the nearby boundary, which suggests that recruitment of neutrophils will be more frequent from the closest blood vessels - a mechanism that can potentially contribute to a faster and a more localized IR.

The physiological significance of each of the above profiles might be determined by e.g. vascularization properties of the tissue as well as the severity of the infection. Thus if the source of infection is small a single wave might be enough to attract a sufficient amount of neutrophils, whereas a larger damage would benefit from repetitive and more frequent waves. In fact this tendency *is *experimentally observed during IR to myocardial injury [10]: In rodent models of myocardial infarction, the cytokines, IL-1, TNF and IL-6 are upregulated up to 50 fold within first hours. They can return to baseline levels if the infarction is small or, if the infarction is large, there is either sustained cytokine upregulation or a second wave of cytokine upregulation [30,31].

## Conclusions

The ability of sending information from one point in space to another is crucial for multicellular organisms, and biological systems have developed many different strategies that address this challenge.

In case of inflammation, the information about the insult - i.e. inflammatory cytokines - can be carried either *passively*, i.e. through diffusion or *actively *amplified by tissue cells. An active transmission of the inflammatory signal is supported by numerous experimental observations [10,32,33] (e.g., local RNA transcription and translation is required for efficient neutrophil emigration [32]).

We here show that the characteristics of the NF-*κ*B regulatory network - fast spatially coupled positive feedback combined with slow negative feedback - are necessary for active propagation of the cytokine wave. Additionally, the characteristic short half-life of cytokines and the recently discovered threshold in NF-*κ*B activation in single cells, are both conditions favoring emergence of the cytokine waves.

While there exists extensive literature on mathematical models addressing the role of the multiple negative feedbacks in NF-*κ*B dynamics [17-19,27,34-36], the positive cytokine feedback has only been considered by Werner et al. [37]. To our knowledge, this is the first time that mathematical modeling addresses the role of cytokine positive feedback in the context of spatially distributed cells.

The cessation or "resolution" of the inflammatory response is as important as its initiation. While moderate and appropriately timed inflammatory response is beneficial - excessive, delayed or prolonged inflammation was shown to be a primary cause in many inflammatory diseases [2,38]. Furthermore, in cases such as tuberculosis, it is the host inflammatory response and not bacterial toxins that are responsible for the damage to the host. In this regard, both the transient nature of cytokine waves and the resulting transient neutrophil recruitment (also observed experimentally [16,33,39-41]) are the mechanisms that naturally minimize inflammatory tissue damage. Additionally, the presence of an obligate "refractory period" following the wave and lasting 5-12 hours will impose further constraints on the IR. Furthermore, our results in Figure 4 showing the persistent IR to transient damage predict that the chronic inflammation ("locked" region) will be accompanied by the re-current acute inflammation ("oscillatory region" close to the blood vessels). Remarkably, acute and chronic inflammation do coexist over long periods in such diseases as rheumatoid arthritis, asthma, multiple sclerosis etc. [2].

The accumulated experimental evidence together with our modeling results suggest that 1) NF-*κ*B is a strong candidate for a mechanism generating "propagating waves" of chemoattractant cytokines. 2) The mechanism behind the propagating waves can have both beneficial and deleterious effects. While it assures reliable signal propagation and avoids long-lasting exposure to toxic cytokines, there are special conditions when the system over-reacts and generates situations which can be interpreted as inflammatory dysfunction as e.g. chronic inflammation.

## Methods

The tissue cells are modeled as discrete units all containing NF-*κ*B-like circuits consisting of the variables N, R and T, which influence each other as sketched in Figure 2E. The interactions are modeled by the following differential equations:(1)(2)(3)

N is activated by T and inhibited by R. The *T *→ *N *activation term is proportional to the amount of in-active NF-*κ*B, (1 - *N*), and the Hill function, , which ensures that activation only occurs when T exceeds a certain activation threshold. Here T is normalized relative to its activation threshold which is thus given by T* = 1. The *R *⊣ *N *inhibition term is proportional to R and is assumed to be saturated in N (equation 1). See additional file 1 for details on the choice of parameters and normalization of the variables. In equation (2) R is activated by N (*N *→ *R *activation term is proportional to N) and decays with the half-life 1/*k_bi_*. Finally, in equation (3), T is activated by N and decays with the half-life *τ_T _*. The *N *→ *T *activation term is modeled with the Hill function, , since NF-*κ*B was reported to form dimers, but the results hold if it is replaced by a term linear in *N*.

The system is induced by the small stimulus, *S*, which represents a small influx of T. In-vivo this influx could correspond to cytokines secreted by macrophages or bacterial endotoxin. We have used *S *= 5 · 10 ^-4^*h*^-1 ^and this influx of T is only added at the site of infection (black dot in Figure 2F) and only at times *t *≥ 0. To account for T diffusion between cells, we add a diffusion term to equation (3), which then becomes:(4)

The distance between the tissue cells is set to *δx *≈ 15*μm *corresponding approximately to the cell size. We set , a numerical value estimated for the diffusible factors of similar size [42] (we have also tested that our main results hold against several fold variation in *D*). The cytokine half-life in bloodstream has been experimentally measured to range between 3-15 minutes [25,26] and was estimated to be 25 minutes in [27]. As short cytokine half-life promotes wave propagation, we use a conservatively long *τ_T _*= 25 min. The results are qualitatively unchanged in the entire range of *τ_T _*= 3 - 25 min.

The parameters for the negative feedback were chosen to be such that the output qualitatively reproduces the transient dynamics of NF-*κ*B, i.e. peak around 30 minutes and the response is decreased by 1 hour; *k_a _*= *k_ai _*= *k_b _*= 5*h*^-1 ^and *k_bi _*= 0.5*h*^-1^. The parameter *K_N _*= 0.3 is chosen relatively to the peak-hight of *N *which can maximally obtain the value *N *= 1 (See normalization in additional file 1). The Hill coefficient *H *= 2 used in the term  is chosen because NF-*κ*B is known to form dimers. The model however also works if we use a simple linear regulation. Thus the only free parameter remaining is the strength of positive feedback, p.

### Generating the alternative scenarios, considered in Figure 1A and 1B

In the scenario seen in Figure 1A there is no regulation of T. The system is modeled by a single differential equation:(5)

In the scenario seen in Figure 1B, T is amplified by N, but there is no transient response of N-corresponding to no R. The system is modeled by the two differential equations:(6)(7)

All results are found using 4th-order Runge-Kutta integration. In Figures 1C, 2F, 3 and 4 we modeled a row of 400 cells and always add the stimulus S, to the middle cell.

## Competing interests

The authors declare that they have no competing interests.

## Authors' contributions

AT conceived the project; PY, BM, AT, MHJ and SK designed the research and wrote the manuscript; PY, BM and AT performed the research. All authors have read and approved the manuscript.

## Supplementary Material

Additional file 1 **Supplementary results**. In this file we provide the details of the model derivation and its robustness to modifications. Please use Acrobat Reader to open this file. Click here for file

## Aknowledgements

We would like to thank Ian Dodd, Sebastian Bernardsson, Kim Sneppen, Namiko Mitarai and Philip Merksamer for the discussions and critical reading of the manuscript.
