# Supplementary material for: Modeling the NF-κB mediated inflammatory response predicts cytokine waves in tissue
Source: BMC Syst Biol. 2011 Jul 19;5:115. doi: 10.1186/1752-0509-5-115 (PMC3152534; doi:10.1186/1752-0509-5-115)
Supplement: Additional file 1 — Supplementary results. In this file we provide the details of the model derivation and its robustness to modifications. Please use Acrobat Reader to open this file. [file 1752-0509-5-115-S1.PDF]

Supplementary Material

Setting up the model

Upon response to external stimuli NF-κB is translocated into the nucleus, where it induces transcription of many proteins including transcription of its own inhibitors. Upon translation these inhibitors bind NF-κB and transports it out of the nucleus. As in all previous models [3, 2, 1, 7], we assume the total amount of NF-κB to be constant, is thus equal to the sum of nuclear, N, and cytoplasmic, N_{cyto} NF-κB at any time : $N_{total} = N + N_{cyto}$. Modeling the tissue cells with NF-κB-like circuits, we have the following equations:

$$\frac{dN}{dt} = k_a \frac{T^3}{T^3 + K_S^3}(N_{total} - N) - k_{ai} R \frac{N}{N + K_R} \tag{1}$$

$$\frac{dR}{dt} = k_b N - k_{bi} R \tag{2}$$

$$\frac{dT}{dt} = p \frac{N^2}{N^2 + K_N^2} - \frac{T}{\tau} - D \frac{\partial^2 T}{\partial x^2} \tag{3}$$

where N resembles NF-κB, R is a "regulator" which captures the effect of numerous inhibitors (both those acting directly on NF-κB, i.e. IkB-family and upstream of it, i.e. A20-family) and T is cytokines – denoted T because we have considered TNF as our main example when constructing the model.

The Hill function $\frac{T^3}{T^3+K_S^3}$ ensures that N is only activated when T exceeds a certain activation threshold, here represented by the parameter K_S. To obtain a clear threshold effect we have chosen to use a Hill coefficent $H = 3$. The model also works with a Hill coefficient $H = 2$ or with a higher Hill coefficient $H > 3$.

The Hill coefficient $H = 2$ used in the term $\frac{N^2}{N^2+K_N^2}$ is chosen because NF-κB is known to form dimers (and N denotes the monomer concentration). The model however also works if we use a simple linear regulation.

The parameters involved in the interactions between N and R, i.e. k_a, k_{ai}, k_b and k_{bi}, where chosen such that NF-κB-response to external stimulation would peak around 30 minutes after stimulation and have decreased back to lower levels after approximately 1 hour. The optimal K_R-value we could choose in order to obtain such dynamics was very small - and hence we choose to simplify the equation for N setting $K_R << N$:

$$\frac{dN}{dt} = k_a \frac{T^3}{T^3 + K_S^3}(N_{Total} - N) - k_{ai} R \tag{4}$$

$$\tag{5}$$

which is similar to saying that the inactivating reaction of N is saturated in N.

Rescaling

The variables in the model are rescaled in the following way:

$$\tilde{N} = N/N_{Total} \tag{6}$$

$$\tilde{T} = T/K_S \tag{7}$$

Substituting the rescaled variables into the equations, gives:

$$\frac{d\tilde{N}}{dt} = k_a \frac{\tilde{T}^3}{\tilde{T}^3 + 1}(1 - \tilde{N}) - k_{ai} R \tag{8}$$

$$\frac{d\tilde{I}}{dt} = \tilde{k}_b \tilde{N} - k_{bi} R \tag{9}$$

$$\frac{d\tilde{T}}{dt} = \tilde{p} \frac{\tilde{N}^2}{\tilde{N}^2 + \tilde{K}_N^2} - \frac{\tilde{T}}{\tau} + D\frac{\partial^2 \tilde{T}}{\partial x^2} \tag{10}$$

with the new parameters:

$$\tilde{k}_b = k_b N_{Total} \tag{11}$$

$$\tilde{p} = p/K_S \tag{12}$$

$$\tilde{K}_T = K_N/N_{Total} \tag{13}$$

Stability analyses

Slow inhibitor dynamics and bistability in fast variables are the two main signatures of the excitable system and these features are also characteristic to Nfkb-like circutis with fast positive feedback coupled to adaptive negative feedback. To understand the system in greater detail we analyze phase-planes of two fast variables, N and T, see Figure 1.

In Figure 1D we see that the system is indeed is bistable. Prior to stimulation the system is in a resting state corresponding to the low T/N stable fixed point. A strong enough stimulus, S, will shift T-nullcline to the right so that only one – high T/N – stable attractor remains. Thus the system is excited in Figure 1. As N and T increase in time and move towards the high T/N attractor, the slow inhibitor, R, catches up and modifies the phase-space by lowering N-nullcline and thus decreasing the high attractor, as shown in Figure 1E. Eventually – at high R – the system is no longer bistable, and has only one – low N/T attractor thus concluding the adaptive response in T.

Shortly after the response occurs, the region becomes transiently insensitive to further perturbations, see interval E'-D' in Figure 1C, after which it relaxes back to the resting state, where it is again sensitive to perturbations. In our model this refractory time is given by parameter k_{bi} and is essentially the timescale of the change in inhibitor, R.

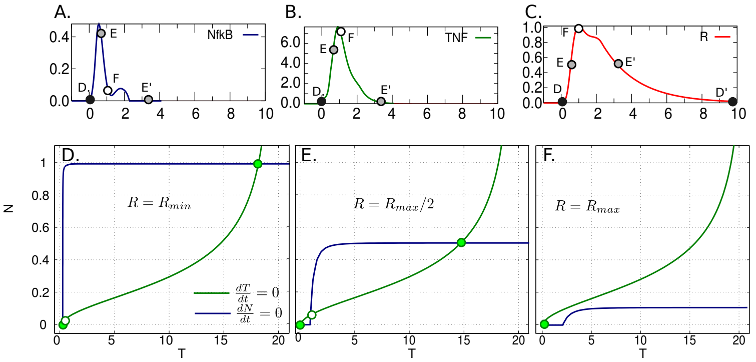

Figure 1: Phase-plane analysis of the model. Time courses of the N, T and inhibitor R A)-C) and the phase spaces corresponding to low, $R = R_{min}$ D), intermediate, $R = R_{max}/2$, E) and high $R = R_{max}$, F) levels of inhibitor R. Stable and unstable fixed points are marked by correspondingly filled and empty green circles. When inhibitor levels, R, are low, system has two fixed points– one low, resting state, and the other one is high, excited state, denoted by filled circles in D). Large enough perturbation from resting state will drive the system to high fixed point. Observe that as R increases, in E), the high fixed point is decreasing, while unstable fixed point increases eventually undergoing saddle node bifurcation (not shown). Eventually – at high enough R, in F) – the system reaches the state where there is only one – low – fixed point, thus returning to resting state where it can be re-excited again.

The wave can propagate in space if a localized signal can excite neighboring regions above the threshold before being damped out. In other words, the time scale of stimulation should be faster than that of inhibition. This separation of time-scale is a key factor required for wave propagation and underlies the mechanism of many types of traveling waves, including those of Belousov-Zhabotinsky (BZ) reaction, the developmental waves of Myxococcus xanthus and Dictyostelium discoideum cells [6].

Interestingly, the phase-plane analysis in Figure 1A-B shows that the particular behavior of the fixed points as function of R is not sensitive to the details of the shape of N-nullcline and T-nullcline. This implies that the excitability criteria do not depend on the details of how N upregulates T, and the hill function $\frac{N^2}{N^2+K_N^2}$ can be replaced with a linear term in N. We have further explored robustness of our model to variations in Hill coefficients and found that we can also reduce Hill coefficent to H=2 in Equation 1, Figure 1A (main text).

The wave can propagate across weakly absorbing regions (small blood vessels)

In principle the bloodvessels are everywhere in the tissue and can be separated by just a few cells. Due to the branching nature of the vascular system, most

of the tissue volume is covered by small blood vessels, where the blood flow is slower and thus cytokines are absorbed at a slower rate.

To test weather the cytokine waves are able to propagate across smaller bloodvessels, we have tested the model in presence of weakly absorbing regions withing the tissue. This additional absorption of T is modeled by reducing half-life of T in equation (4). See Figure 2.

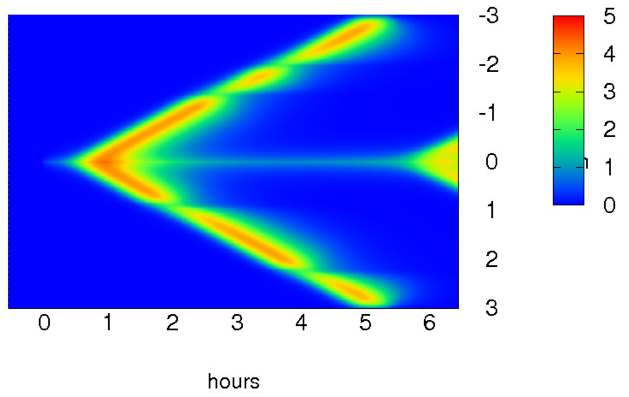

Figure 2: Exploring model robustness. Out of 400 cells we chose 4 groups of 5 cells each to represent small blood vessels. We modeled the effect of weakly absorbing regions by setting T to have a considerably shorter half-life, $\tau_T =$ 5min.

More detailed, nested feedback model, generates propagating wave

To test weather our simplified NF-κB-like circuits give the same result as a more detailed NF-κB model we have modeled the NF-κB network sketched in Figure 3. In this model the inhibitors IκB$_{\alpha,\epsilon}$ and A20 are modeled explicitly, and we confirm that this model gives similar results - namely propagating cytokine waves. The model was simulated with parameters similar to those used in [5], and the only changes we have made to the original model is to include the positive feedback between T and N - hence introducing the parameter p. Our results show that this model exhibits a similar dependence on p, and that there exists regions of re-emerging waves.

Chemotaxis

In order to obtain the neutrophil-trajectories, which we show in Figure 1, we have constructed a "Local excitation - global inhibition" model [4]. The neutrophil-"body" is divided the into compartments, which allows for the cell to respond locally. For simplicity we have chosen to model only two compartments

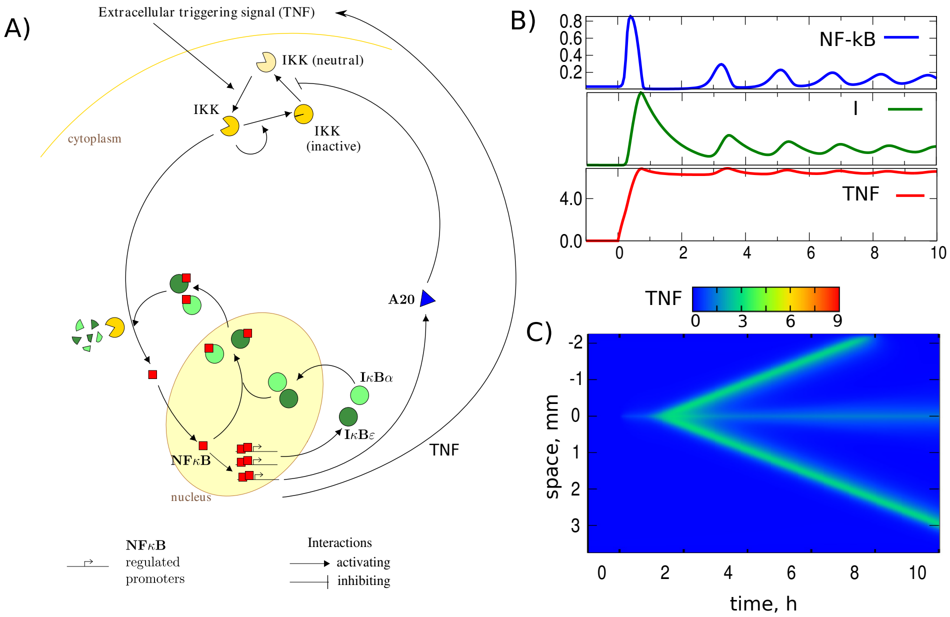

Figure 3: A) Nested feedback model of NF-κB dynamics in single cells, consisting of positive TNF feedback that allows signal amplification and negative feedbacks that captures NF-κB transient dynamics [5]. B) Single cell dynamics, with blue line showing NF-κB, green line, the dynamics Iκb inhibitors (Iκbα + Iκbϵ) and corresponding TNF profile. C) Nested feedback circuits couple in space produce propagating wave profile.

- front and back. The model can easily be extended into several compartments, which would correspond to a cell with a higher spatial resolution.
A chemoattractant-gradient is sensed by neutrophils, through cytokine binding to receptors. Activated receptors, induce pseudopod formation locally - hence inducing movement proportional to the net distribution of activated receptors. Activated receptors also induce production of a global inhibitor, a molecule that can diffuse fast through the cell, bind and inactivate both free and occupied receptors.
The dynamics of free,R_f, activated,R_a, and inhibited, R_i, receptors (within each compartment) can be described by the equations:

$$\frac{R_f}{dt} = -(k_{fa}T + k_{fi}I_G)R_f + k_{af}R_a + k_{if}R_i \quad (14)$$

$$\frac{R_a}{dt} = k_{fa}TR_f - k_{af}R_a \quad (15)$$

$$\frac{R_i}{dt} = k_{fi}I_GR_f - k_{if}R_i \quad (16)$$

The dynamics of the global inhibitor, I_G, is described by the equation:

$$\frac{dI_G}{dt} = C\frac{\sum_j R_{a,j}}{\sum_j R_{a,j} + 1} - I_G/\tau_G \tag{17}$$

where j runs over all compartments. The velocity of the immune cell is modeled to be proportional to the net gradient of activated receptors. Using a model with two compartments the velocity is calculated in the following way:

$$vel = A\Delta R_{a,j} \tag{18}$$

where the last equality sign holds for the case with only two compartments.

We have chosen the parameters such that they match with the width and speed of the cytokine waves, and we have used the values: $k_{fa} = 1$, $k_{af} = 2$, $k_{if} = 2$, $k_{fi} = 20$, $A = 10000$, $C = 83.88$, $\tau_G = 0.012$.

References

[1] Louise Ashall, Caroline A Horton, David E Nelson, Pawel Paszek, Claire V Harper, Kate Sillitoe, Sheila Ryan, David G Spiller, John F Unitt, David S Broomhead, Douglas B Kell, David A Rand, Violaine Se, and Michael R H White. Pulsatile stimulation determines timing and specificity of nf-kappab-dependent transcription. Science, 324(5924):242–246, Apr 2009.

[2] Alexander Hoffmann, Andre Levchenko, Martin L Scott, and David Baltimore. The ikappab-nf-kappab signaling module: temporal control and selective gene activation. Science, 298(5596):1241–1245, Nov 2002.

[3] Sandeep Krishna, Mogens H Jensen, and Kim Sneppen. Minimal model of spiky oscillations in nf-kappab signaling. Proc Natl Acad Sci U S A, 103(29):10840–10845, Jul 2006.

[4] Lan Ma, Chris Janetopoulos, Liu Yang, Peter N Devreotes, and Pablo A Iglesias. Two complementary, local excitation, global inhibition mechanisms acting in parallel can explain the chemoattractant-induced regulation of pi(3,4,5)p3 response in dictyostelium cells. Biophys J, 87(6):3764–3774, Dec 2004.

[5] Benedicte Mengel, Sandeep Krishna, Mogens H. Jensen, and Ala Trusina. Theoretical analyses predict a20 regulates period of nf-kb oscillation. arxive[physics.bio-ph], 0911.0529, Nov 2009.

[6] B. M. Sager. Propagation of traveling waves in excitable media. Genes Dev, 10(18):2237–2250, Sep 1996.

[7] Sava Tay, Jacob J Hughey, Timothy K Lee, Tomasz Lipniacki, Stephen R Quake, and Markus W Covert. Single-cell nf-kappab dynamics reveal digital activation and analogue information processing. Nature, 466(7303):267–271, Jul 2010.
